# Two Types of Antibodies Are Induced by Vaccination with A/California/2009pdm Virus: Binding near the Sialic Acid-Binding Pocket and Neutralizing Both H1N1 and H5N1 Viruses

**DOI:** 10.1371/journal.pone.0087305

**Published:** 2014-02-05

**Authors:** Nobuko Ohshima, Ritsuko Kubota-Koketsu, Yoshitaka Iba, Yoshinobu Okuno, Yoshikazu Kurosawa

**Affiliations:** 1 Division of Antibody Project, Institute for Comprehensive Medical Science, Fujita Health University, Toyoake, Aichi, Japan; 2 Department of Virology, Research Institute for Microbial Diseases, Osaka University, Suita, Osaka, Japan; 3 The Research Foundation for Microbial Diseases, Osaka University, Kanonji, Kagawa, Japan; Chinese Academy of Medical Sciences, China

## Abstract

Many people have a history of catching the flu several times during childhood but no additional flu in adulthood, even without vaccination. We analyzed the total repertoire of antibodies (Abs) against influenza A group 1 viruses induced in such a flu-resistant person after vaccination with 2009 H1N1 pandemic influenza virus. They were classified into two types, with no exceptions. The first type, the products of B cells newly induced through vaccination, binds near the sialic acid-binding pocket. The second type, the products of long-lived memory B cells established before vaccination, utilizes the *1-69* V_H_ gene, binds to the stem of HA, and neutralizes both H1N1 and H5N1 viruses with few exceptions. These observations indicate that the sialic acid-binding pocket and its surrounding region are immunogenically very potent and majority of the B cells whose growth is newly induced by vaccination produce Abs that recognize these regions. However, they play a role in protection against influenza virus infection for a short period since variant viruses that have acquired resistance to these Abs become dominant. On the other hand, although the stem of HA is immunogenically not potent, the second type of B cells eventually becomes dominant. Thus, a selection system should function in forming the repertoire of long-lived memory B cells and the stability of the epitope would greatly affect the fate of the memory cells. Acquisition of the ability to produce Abs that bind to the stable epitope could be a major factor of flu resistance.

## Introduction

Influenza is an infectious disease of the respiratory tract that affects millions of people every year. Since antibodies (Abs) play important roles in protection against influenza virus, preventive vaccination has been one of the most efficient measures of influenza control. Hemagglutinin (HA), which is the main target for virus-neutralizing Abs, mediates virus entry into cells at two steps [1]. First, HA binds to the cell receptor, sialic acid. After internalization of viruses by endocytosis, HA undergoes a drastic conformational change induced by low pH. Neutralizing Abs have one of the following activities: prevention of the binding reaction between HA and sialic acid [2] and prevention of low-pH-induced conformational change of HA [3]. The former binds to the site near the sialic acid-binding pocket on the globular head in HA1, and the latter binds to the stem region formed mainly by HA2. Since the dominant immune response is the first type and mutations can be easily introduced into the target sites without losing the receptor-binding activity, variant viruses that have acquired resistance to these Abs become dominant and cause annual epidemics. Historically, it was long believed that all effective neutralizing Abs are the first type; therefore, vaccine strains should be changed almost every year to remain effective. As long as the vaccine strain is a good match to the circulating virus, vaccination is effective for preventing virus infection.

The mode of response against influenza virus infection is very heterogeneous among the human population. For example, many people who have experienced several influenza infections in their childhood have not experienced additional influenza infections, even without vaccination. There has been no report clearly showing what kinds of ability such flu-resistant people obtained through repetitive influenza infections in their childhood. In 2009, a swine-origin H1N1 influenza virus (S-OIV) emerged and spread rapidly among the human population, resulting in its classification as the first pandemic of the 21st century [4]. It has been generally believed that humans lack immunity to the newly appearing influenza virus at the outbreak of a pandemic because they are naive to the virus. In the case of S-OIV, however, long-lived memory B cells that produce broadly neutralizing Abs not only against seasonal H1N1 viruses but also S-OIV are found in many elderly individuals [5]–[7].

To analyze the total repertoire of neutralizing Abs against influenza viruses in humans, we developed the following experimental strategy in our previous study [8]. A large number of B lymphocytes are collected by apheresis from a donor, and a huge Ab library is constructed by using phage-display technology. The library is screened with formalin-treated virus particles. The clones that have both binding activity and neutralizing activity to the virus are isolated and their strain specificity is extensively characterized. Although only B lymphocytes that are circulating in peripheral blood are analyzed in our experimental system, the results appeared to represent the total repertoire of neutralizing Abs formed in the donor's body [8]. In the present study, we applied this strategy to the analyses of Abs that are present in the human body before and after vaccination with a newly appearing kind of virus. Since S-IOV newly emerged, the results should reflect only the effects of vaccination without the effects of a natural infection with living S-IOV.

## Materials and Methods

### Viruses

The following influenza viruses were used in this study. A/H1N1pdm: A/California/7/2009pdm (Cal09), A/Suita/1/2009pdm (Sui09); A/H1N1: A/New Caledonia/20/1999 (NC99), A/Solomon Islands/3/2006 (SI06), A/Brisbane/59/2007(Bri07). A/H3N2: A/Panama/2007/1999. A/H5N1: A/Indonesia/5/2005/PR8-IBCDC-RG2. Abbreviations for the strains are shown in parentheses.

### Ethics statement

Ethical approval was granted by the Research Ethics Committee of Fujita Health University. Signed informed consent was obtained from the blood donor.

### Construction of Ab library

Phage Ab libraries were constructed as described previously [8]. Briefly, mononuclear cells from a donor born in 1947 were collected by apheresis from the equivalent of 3 litters of blood before and after vaccination. The cells included 8.0×10^8^ B lymphocytes (before vaccination) and 1.2×10^9^ B lymphocytes (after vaccination). Large combinatorial Ab libraries were constructed by using the phage-display method as described previously [9]. The size of the libraries were: before vaccination, 1.6×10^9^ clones for heavy (H) chain, 2.0×10^9^ clones for light (L) chain and 1.4×10^10^ clones for Fab; after vaccination, 3.2×10^9^ clones for H chain, 1.3×10^9^ clones for L chain and 2.6×10^10^ clones for Fab.

### Screening of the library

Phages bound to virus particles were selected by a panning method as described previously [10]. In brief, formalin-treated virus particles of Cal09 or Bri07 strains were used as antigens (Ags) in the screenings. After two and three pannings, *E. coli* (DH12S) cells were infected with the eluted phages and spread onto the LB plates containing 100 µg/ml ampicillin and 0.2% glucose. *E. coli* colonies harboring phagemid were picked up and grown in 2× YT medium containing 100 µg/ml ampicillin, 0.05% glucose and 1 mM isopropyl-β-D-thiogalactopyranoside at 30°C overnight. During growth of *E. coli*, the Fab-cp3 form of Ab was secreted into the medium [11]. The culture supernatants containing Fab-cp3 molecules were subjected to enzyme-linked immunosorbent assay (ELISA) against H1N1 virus used as Ag in the screening and H3N2 virus. Clones that bound only to H1 were selected and subjected to further analyses.

### ELISA

Formalin-treated virus particles were coated onto 96 well Maxisorp immunoplates (Nunc), and Fab-cp3 Ab in the supernatant of *E. coli* culture was added to each well. After incubation with rabbit anti-cp3 Ab (MBL), the wells were further incubated with peroxidase-conjugated goat anti-rabbit IgG (H+L chain; MBL). Then, HRP substrate (OPD; Wako) was added to each well, and the color of the sample was developed. After stopping the peroxidase reaction by adding H_2_SO_4_, the absorbance of the sample at 492 nm was measured.

### Sequence analysis

The nucleotide sequences of V_H_ fragments of isolated Ab clones were determined by using GenomeLab Dye Terminator Cycle Sequencing with Quick Start Kit (Beckman Coulter) and a CEQ2000 DNA Analysis System (Beckman Coulter). The T7ETZ (5′-TAATACGACTCACTATAGGG-3′) was used as the V_H_ sequencing primer.

### Virus neutralization test

For measurement of virus neutralizing activity, a focus reduction assay [12] was performed by using the single cycling (VN) or multiple cycling (M-VN) method. Two hundred or 500 µg/ml of Fab-PP Abs (P denotes a single Fc-binding domain of protein A) or two-fold serial dilutions of serum were mixed with an equal volume of 100 FFU of influenza virus and applied to MDCK cells in a 96-well plate. In the VN method, after incubation with the mixture, the cells were washed with serum-free MEM and cultured in MEM containing 0.4% BSA at 37°C for 15 h. In the M-VN method, after incubation, MEM containing 0.4% BSA, 5 µg/ml of acetylated trypsin, and 0.5% methyl cellulose of equal volume to the mixture was further added to the cells without removing the mixture and the cells were incubated at 37°C for 28 h. Then, the cells were fixed with ethanol and stained with PAP (peroxidase and anti-peroxidase) complex. The number of foci containing one and more cells (VN method) or four and more cells (M-VN method) was counted. The results were indicated as the focus reduction rate (%) for Fab-PP Ab or the reciprocal of the highest dilution of serum to show 50% focus reduction rate for serum.

### Hemagglutination inhibition (HI) assay

The HI test was performed as described previously [12]. In brief, serial dilutions of 160 µg/ml of purified Fab-PP or donor's serum in PBS were prepared. Serial dilutions of Fab-PP or serum were preincubated with 4 HA units of virus per well. Guinea pig red blood cells (0.75%) in PBS were added to each well, and the plate was incubated at room temperature for 30–60 min. The results were shown as the lowest concentration (µg/ml) of Fab-PP Ab or the reciprocal of the highest dilution of serum to inhibit hemagglutination.

### Competition ELISA

Competition ELISA was performed by using the Fab-PP form of Ab [13] or C179 [3] for detection of the binding activity to virus particles and the Fab-cp3 form of Ab as a competitor. Fab-cp3 molecules in the supernatant of *E. coli* culture were concentrated 20-fold before use. Formalin-inactivated virus particles were coated onto a 96 well Maxisorp immunoplate. A total of 50 µl of Fab-PP at an optimized concentration was mixed with 50 µl of 20-fold concentrated Fab-cp3 and the mixture was added to a virus-coated well. Then, peroxidase-conjugated rabbit anti-streptavidin Ab was added to each well as a secondary Ab. When C179 at the final concentration of 0.25 µg/ml was used for detection of the binding activity to virus strain, each well was incubated with peroxidase-conjugated goat anti-mouse IgG (H+L chain; MBL) as a secondary Ab. Then, HRP substrate (OPD; Wako) was added to each well, and the color of the sample was developed. After stopping the peroxidase reaction by adding H_2_SO_4_, the absorbance at 492 nm was measured.

### Preparation of mAb specifically bound to 1-69Abs

Five kinds of Fab-PP form of 1-69Ab, F081-007, F083-103, F083-115, F083-305, and F083-311 were purified and injected into the foot-pad of Balb/c mice with Freund's complete adjuvant once and 11 days later without adjuvant once more. Three days later after the second injection, the lymphocytes were isolated from the inguinal lymph node and fused with the myeloma line P3-X63AG8.653. After cloning of hybridomas, the culture media were screened using the above 1-69Abs and various kinds of Abs that do not utilize *1-69* V_H_ gene by ELISA. The clones that bind to multiple kinds of 1-69Ab but do not bind to any of non 1-69Abs were selected. This part of the work was performed by MAB Institute Inc. (Sapporo, Japan).

### Determination of the serum concentration of IgG that uses the *V_H_1-69* germline gene

A human IgG ELISA Quantitation set (Bethyl Laboratories, Inc.) was used, with slight modification. K1-18 Ab for detection of IgG using *V_H_ 1-69* germline gene and affinity-purified Human IgG Coating Antibody for standard curve were coated onto a 96-well Maxisorp immuonoplate. Serum or human reference serum (for standard curve) was added to the assigned well. After incubation with HRP-conjugated Human IgG Detection Antibody, TMB substrate was added to each well. The peroxidase reaction was stopped by adding H_2_SO_4_, and the absorbance of sample at 450 nm was measured. The concentration of IgG bound to K1-18 Ab was calculated from the standard curve of human reference serum.

### Virus-neutralizing activity of serum in the presence of K1-18 Ab

The virus neutralization test described above was modified. In brief, serum treated with receptor destroying enzyme (RDE) was diluted at 1∶10 or 1∶20 in serum-free medium and mixed with an equal volume of 800 or 1,600 µg/ml of K1-18 Ab. After incubation, two-fold serial dilutions of the mixture were mixed with an equal volume of 100 FFU of influenza virus and applied to MDCK cells in a 96-well plate. After incubation, the cells were fixed with ethanol and stained with PAP (peroxidase and anti-peroxidase) complex. The reciprocal of the highest dilution of serum to show a 50% focus reduction rate was indicated as virus neutralizing activity.

### Binding activity of K1-18 Ab to 1-69Abs

Ten µg/ml of Fab-PP form of 1-69Ab was coated onto 96 well Maxisorp immunoplates (Nunc), and K1-18 Ab was added to each well. After incubation with peroxidase-conjugated goat anti-mouse IgG (H+L chain, MBL), HRP substrate (OPD; Wako) was added to each well. After stopping the peroxidase reaction by adding H_2_SO_4_, the absorbance of the sample at 492 nm was measured.

### Binding activity of Abs to virus particles under the presence of K1-18 Ab

Formalin-inactivated virus particles (Bri07) were coated onto a 96 well Maxisorp immunoplate. Fab-cp3 molecule that was diluted from 20-hold concentrated solution at an optimized concentration was mixed with an equal volume of 200 or 400 µg/ml of K1-18 Ab and incubated at 37°C for 1 h. The mixture was added to virus coated well and the wells were incubated at 37°C for 1 h. After incubation with rabbit anti-cp3 Ab (MBL), the wells were further incubated with peroxidase-conjugated goat anti-rabbit IgG (H+L chain; MBL). Then, HRP substrate (OPD; Wako) was added to each well, and the color of the sample was developed. After stopping the peroxidase reaction by adding H_2_SO_4_, the absorbance of the sample at 492 nm was measured.

### Nucleotide sequence accession number

The nucleotide sequences of the heavy and light chains of isolated Abs have been deposited in the DDBJ database (accession numbers AB826498 to AB826560 for the heavy chains and AB845357–AB845391 for the light chains).

## Results

### Two types of virus-neutralizing Abs with different characteristics

**Figure 1:**
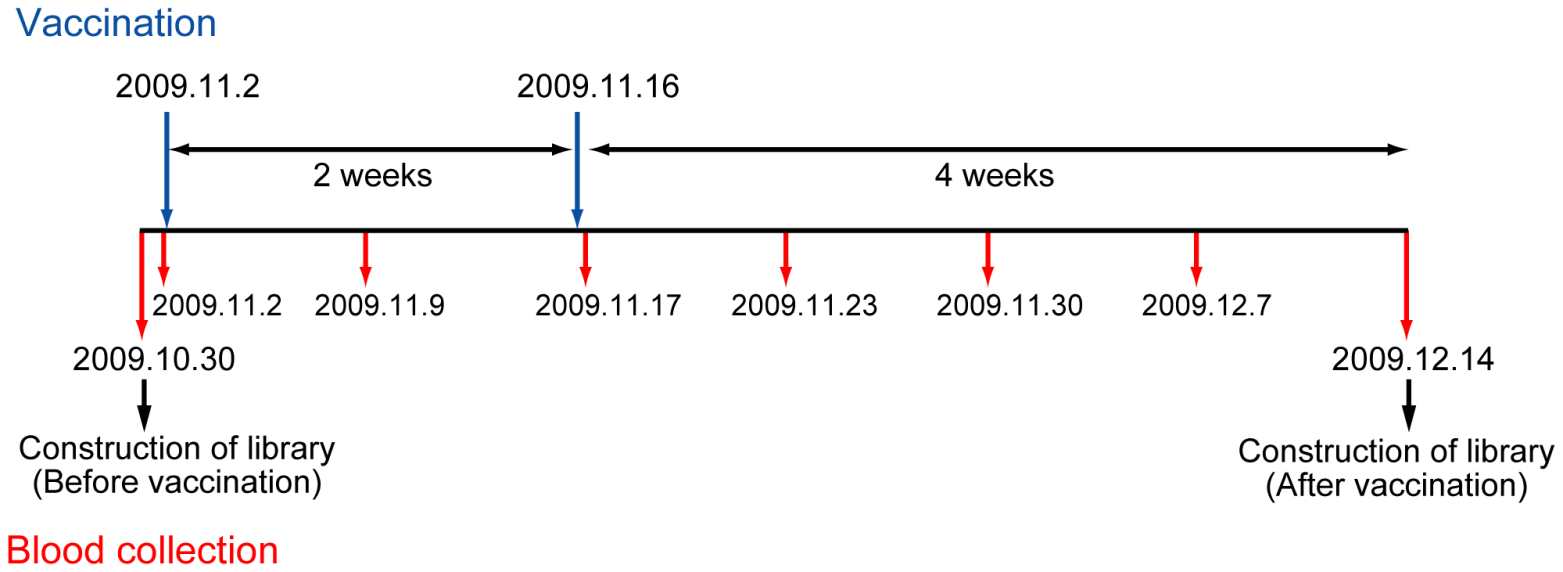
Schedule of vaccination and blood collection. In 2009, a donor born in 1947 was vaccinated with A/California/7/2009 pandemic vaccine strain two times (on November 2 and 16). Blood was collected from the donor two times before vaccination (on October 30 and November 2), once after 1st vaccination (on November 9) and 5 times after 2nd vaccination (on November 17, 23, 30, December 7, and 14). Large phage Ab libraries were prepared from blood collected on October 30 (before vaccination) and December 14 (after vaccination).

We examined the total repertoire of Abs induced by vaccination with S-IOV. The blood donor in this study was born in 1947 and was infected with influenza several times in his childhood (possibly by H1N1 and H2N2) and in 1968 (probably by H3N2). For 41 years afterwards, he did not contract influenza, and he was never vaccinated against influenza. The schedule of vaccination and blood collection is shown in Figure 1. The vaccination and blood collection were performed during the period from late October to mid-December 2009, and the examinee did not have an opportunity to be naturally infected with S-IOV. By using B lymphocytes collected before and after vaccination, two large Ab libraries were constructed and subjected to screenings by panning with pandemic H1N1 (Cal09) and seasonal H1N1 (Bri07) virus particles. After the second and the third rounds of panning with the viruses, 120 clones were isolated. The clones that bound to the H1N1 virus particle that had been used for screenings were further analyzed. The clones that bound to both H1N1 and H3N2 with equal strength were excluded, since it is likely that they were anti-NP Abs [8]. The HA-binding activity of respective clones was further confirmed by Western blot of virus proteins used as Ags in the screenings. Among the 240 clones isolated in respective screenings, the number of clones that were judged to be anti-HA Abs is as follows: screening 1 (after vaccination, with pandemic virus), 105 clones; screening 2 (before vaccination, with pandemic virus), 3 clones; screening 3 (after vaccination, with seasonal virus), 58 clones; and screening 4 (before vaccination, with seasonal virus), 16 clones. V_H_ nucleotide sequences of all these clones were determined. Comparison of the amino acid sequences revealed that these 182 clones were composed of 96 unique monoclonal Abs (mAbs). Based on sequence similarities of V_H_ fragments, the 96 clones were classified into 63 groups. The amino acid sequence of the V_H_ fragment and the nucleotide sequence of complementarity-determining region 3 (CDR3) of all the clones are available in Figures S1 and S2.

**Figure 2:**
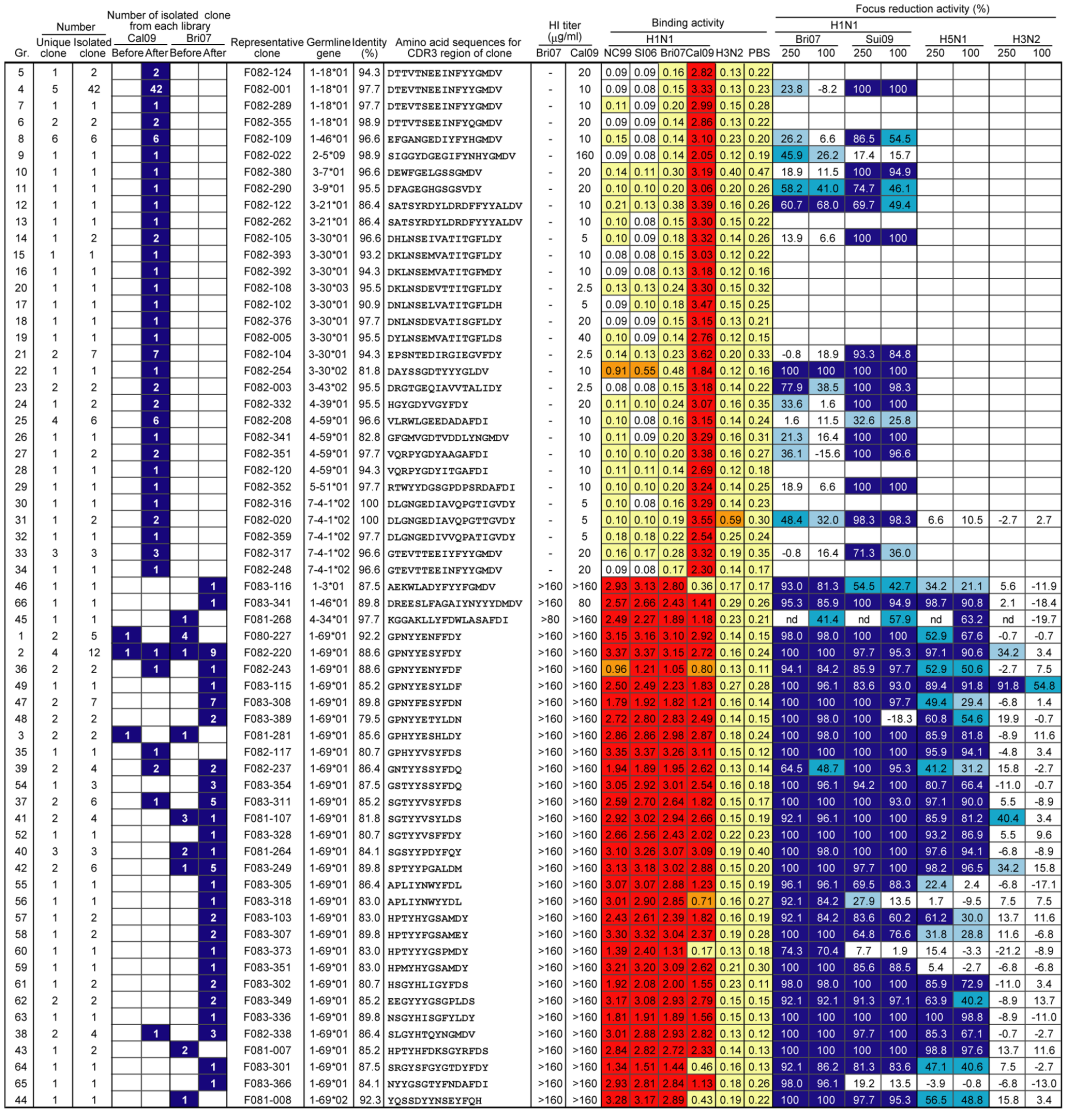
Activities of representative clones classified into 63 groups. The first three columns contain the numbers of clones isolated by the screenings. Germline genes were identified by comparing the amino acid sequence of V_H_ of the representative clone with the sequences of all of the germline V_H_ genes, and the identity (%) is indicated. The amino acid sequence of CDR3 is shown. The binding activity to four H1N1 (NC99, SI06, Bri07 and Cal09) and one H3N2 virus particles was examined by enzyme-linked immunosorbent assay (ELISA). Absorbance at 492 nm is shown as follows: ≥1.00 (red), 0.50–0.99 (orange), and 0.10–0.49 (yellow). The virus neutralizing activity of 100 or 250 µg/ml Fab-PP Ab against H1N1, H5N1, and H3N2 viruses was examined by the focus reduction test. The reduction rate is shown as a percentage as follows: ≥60% (dark blue), 40%–60% (blue), and 20%–40% (light blue). The HI activity of Fab-PP Ab was measured by using two H1N1 (Bri07 and Cal09) viruses. The lowest concentration (µg/ml) of Fab-PP Abs to inhibit hemagglutination is shown.

The HA binding activity, hemagglutination inhibition (HI) activity, and the virus-neutralizing activity [12] of representative clones of the 63 groups were examined. As shown in Figure 2, all of the representative clones were classified into two types with no exceptions. Clones classified as the first type bind only to the pandemic H1N1 and not to the seasonal H1N1. All of these clones show HI activity and were isolated only from the screening 1. Clones classified as the second type bind not only to the pandemic H1N1 but also to all of the seasonal H1N1s. While they do not show HI activity, most of them neutralize not only H1N1 viruses but also H5N1 virus. Judging from the low frequency of mutations, such as 0% to 5%, the majority of the first type of clones should be the products of B cells that have been newly induced through vaccination. On the other hand, all of the second type of clones (except for a few) should correspond to the products of long-lived memory B cells that were established before vaccination, judging from the high frequency of mutations, such as 10% to 15%. Furthermore, they all utilized the *1-69* V_H_ gene, with the exception of three clones.

### Newly appearing Abs after vaccination

**Figure 3:**
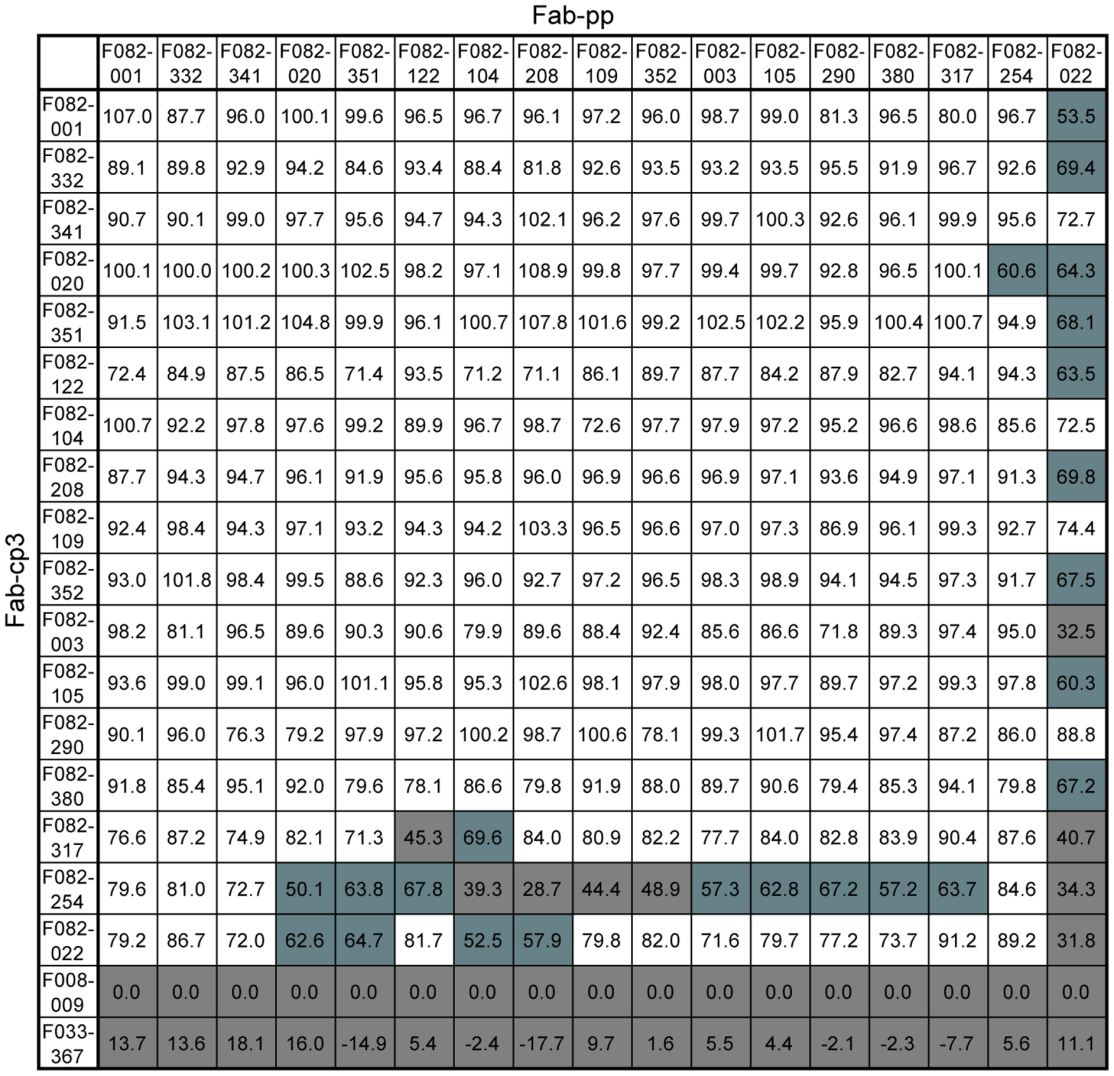
Competitive inhibition of binding to HA among the Abs newly induced by vaccination. The binding activity of Fab-PP Ab (indicated at upper side) to Cal09 virus particles was measured by ELISA under the presence of a 10-times greater concentration of Fab-cp3 Ab (indicated at the left side). F008-009 and F033-367 are not anti-HA Abs and were used as controls. The binding inhibition was calculated as follows: the absorbance value under the presence of F008-009cp3 was used as 100% binding, and the degree of reduction in the absorbance value under the presence of Fab-cp3 of Ab was measured and shown as percent inhibition. Percent inhibition is shown as follows: ≥70% (white), 50%–70% (blue), 0%–50% (grey). The experiment was performed at least three times in duplicate.

Since virtually all the first-type Abs showed HI activity, it is likely that their epitopes are located in the region surrounding the sialic acid-binding pocket. To systematically examine the relative position of the epitopes recognized by these clones, we adopted the competition method that we used in a previous study [14]. While respective mAbs are initially prepared as the Fab-cp3 form after the screening of libraries, they can be easily changed to the Fab-PP form (P denotes a single Fc-binding domain of protein A) in our vector construct [13]. If the epitope recognized by clone A is overlapped with that by clone B, the binding of the Fab-PP form of clone A to HA is largely disturbed by the presence of a large amount of Fab-cp3 form of clone B. Based on this principle, the competition study was performed by using 17 clones selected from the first-type Abs listed in Figure 2. As shown in Figure 3, 14 of the 17 clones competed well against one another for the binding to HA. In the case of three clones, F082-317, F082-254 and F082-022, the degree of disturbance for the binding was low. These observations are consistent with other data. Only F082-022 showed very low HI activity (titer 160 µg/ml). F082-254 binds to HA of not only pandemic virus but also seasonal virus and neutralizes both viruses. Furthermore, a high frequency of mutation, such as 18%, was observed in this clone. In the case of F082-317, the binding activity to HA is high but the neutralizing activity is relatively low.

Based on these observations, we concluded that the sialic acid-binding pocket and its surrounding region on HA are immunogenically very potent and that virtually all of the B cells whose growth is newly induced and expanded by vaccination produce Abs that recognize these regions. We also concluded that when formalin-treated virus particles that are not alive are used as vaccine, B cells producing Abs that are able to bind to HA but not able to neutralize virus are not induced at a substantial level. The second conclusion suggests that the non-neutralizing epitope on HA, even if it exists, is immunologically impotent.

### Abs encoded by long-lived memory B cells

**Figure 4:**
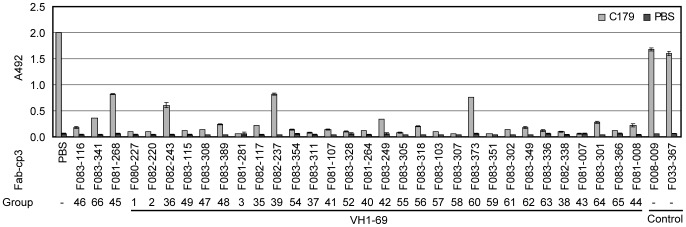
Inhibition of the binding of C179 to HA by type 2 Abs. Binding of C179 to Bri07 virus particles was examined under the presence of a 10-times greater concentration of various Fab-cp3 Abs by ELISA. F008-009 and F033-367 are not anti-HA Abs and were used as negative controls. The group number is indicated under the name of the clone. The experiment was performed two times in duplicate, and the error bars show standard deviation.

While the second type of clones is classified into 32 groups, they all utilized the *1-69* V_H_ gene, with the exception of three clones. Furthermore, the majority of these Abs were able to neutralize not only all H1N1 viruses but also the H5N1 virus. These observations suggest that the epitopes recognized by these clones should be located near the epitope recognized by C179, as shown by several groups [15], [16]. We examined whether the binding of C179 to HA is disturbed by a large excess of the Fab-cp3 form of these clones. As shown in Figure 4, all of the clones including the three that did not utilize the *1-69* V_H_ gene disturbed the binding of C179 to HA. Thus, the second type of clones should bind to the membrane-proximal stem of HA in the same or similar way as other Abs using the *1-69* V_H_ gene (1-69Abs), such as CR6261 and F10, which have already been described by other groups [15], [16]. Figure 4 also indicates that four clones, F081-268, F082-243, F082-237, and F083-373, showed weaker disturbance activity than the others. The data shown in Figure 2 indicate that F081-268 had strong HA-binding activity, but the neutralizing activity was weak. The other three clones showed relatively weak binding and neutralizing activities. Thus, while all of the epitopes recognized by the second type of Abs are not exactly the same as that recognized by C179, we did not find any clone that binds to an epitope totally different from that recognized by C179. This result suggests that this epitope has been shared and stably kept among group 1 viruses. Even if the immunogenicity of this epitope is weak, once humans acquire B cells that produce Abs that recognize this epitope, the B cells could remain in the body as memory cells for a long period and become major players against group 1 viruses. Interestingly, one clone (F083-115) that utilized the *1-69* V_H_ gene is able to neutralize even H3N2 virus.

### Presence of Abs secreted into the serum

Two biological activities, HI activity and virus-neutralizing activity, against three viral strains, H1N1 pandemic virus, seasonal H1N1 virus and H5N1 virus, were measured in eight samples of sera collected from the examinee on different days, as shown in Figure 1. As indicated in Table 1, in the case of HI, the activity against seasonal H1N1 and H5N1 viruses was not detected in any of the sera. HI activity against H1N1 pandemic virus started to appear 2 weeks after the first vaccination. In the case of virus-neutralizing activity measured by standard focus reduction assay, the activity against H1N1 pandemic virus already started to increase 1 week after vaccination and reached a plateau 2 weeks after vaccination. Against seasonal H1N1 virus, virus-neutralizing activity was detected even before vaccination and increased 1 week after vaccination. Against H5N1 virus, virus-neutralizing activity was not detected by the standard focus-reduction assay [12] but was detected by a more sensitive method that is described in Materials and Methods.

**Table 1:** HI and virus neutralizing activity of serum against H1N1 and H5N1 virus strains.

	A/Suita/1/2009H1N1pdm		A/Brisbane/59/2007(H1N1)			A/Indonesia/5/2005(H5N1)		
Date of blood collection	HI^a^	VN50^b^	HI	VN50	M-VN50^c^	HI	VN50	M-VN50
2009.10.30^d^	<40	<40	<40	40	160	<40	<40	ND^e^
2009.11.02^d^	<20	40	<20	20	160	<20	<40	ND
2009.11.09	<20	320	<20	80	160	<20	<40	ND
2009.11.17	80	1280	<20	80	320	<20	<40	40
2009.11.23	40	1280	<20	80	320	<20	<40	40
2009.11.30	40	1280	<20	80	320	<20	<40	40
2009.12.07	40	1280	<20	80	320	<20	<40	40
2009.12.14	40	2560	<40	80	320	<40	<40	ND

a HI activity.

b Virus neutralizing activity detected by VN method.

c Virus neutralizing activity detected by M-VN method.

d Serums before vaccination.

e Not determined.

**Figure 5:**
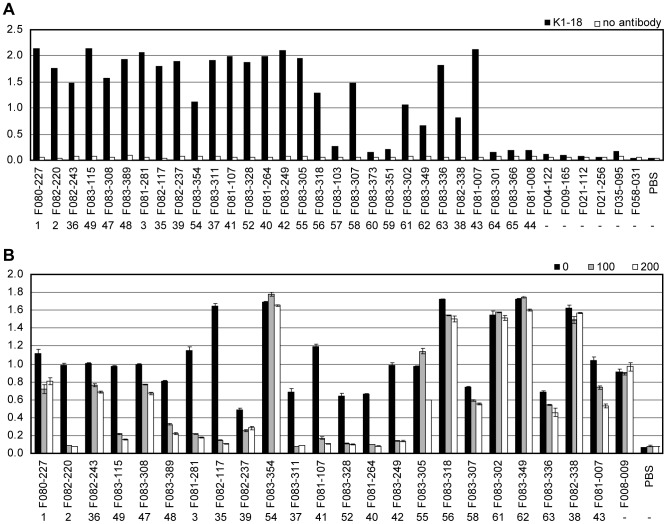
Characterization of K1-18 Ab. (A) Binding activity of K1-18 Ab to 1-69Abs. Binding activity of K1-18 Ab to 1-69Abs was examined by ELISA. F004-122, F009-165, F021-112, F021-256, F035-095, and F058-031 are not 1-69Abs. The group number is indicated under the name of the clone. (B) Binding activity of 1-69Abs to virus particles under the presence of K1-18 Ab. Binding activity of 1-69Abs to Bri07 virus particles was examined under the presence of 100 or 200 µg/ml K1-18 Ab by ELISA. F008-009 is not anti-HA Ab and was used as a negative control. The group number is indicated under the name of the clone. The experiment was performed in duplicate, and the error bars show standard deviation.

To prove that 1-69Abs are really responsible for neutralizing H1N1 pandemic virus, seasonal H1N1 virus and H5N1 virus, we prepared mAb that can specifically bind to 1-69Abs, that is, anti-idiotypic Ab. We expected that the region including two amino acids, isoleucine at the 53rd residue and phenylalanine at the 54th residue, in the V_H_ domain should be immunogenic in mice since the presence of two hydrophobic residues in CDR2 is very unique and found only in 1-69Ab of humans [17]. Furthermore, we presumed that the anti-idiotypic Ab may inhibit the binding of 1-69Ab to HA since these two amino acids are directly involved in the Ab/HA interaction [15], [16]. We successfully isolated mAb K1-18 that can bind to around 80% of 1-69Abs listed in Figure 2 and can inhibit the binding of a half of the 1-69Abs to HA as indicated in Figure 5A and B.

By using K1-18 as a probe, the amount of the IgG form of 1-69Ab in serum was measured. The results indicated that it was present at a concentration of 4.58 µg/ml in the serum collected on October 30 (before vaccination) and increased after vaccination to an concentration of 11.24 µg/ml in the serum collected on December 14. Virus-neutralizing activity against pandemic and seasonal H1N1 viruses was measured in the presence of K1-18. In the case of H1N1 pandemic virus, K1-18 definitely inhibited the neutralizing activity, as indicated in Table 2. Against seasonal H1N1 virus, the inhibition was clearly observed, although not perfectly. Thus, we concluded that 1-69Abs were really present in the sera and functioned in the neutralization of H1N1 viruses. Furthermore, the data in Table 2 suggested that when two types of functionally different Abs co-exist, the first type prevents HA/receptor interaction and the second type prevents low-pH-induced conformational change of HA, and the virus-neutralizing activity synergistically increases.

**Table 2:** Virus neutralizing activity of serum in the presence of K1-18 Ab.

	A/Suita/1/2009H1N1pdm			A/Brisbane/59/2007(H1N1)		
	Concentration of K1-18 (µg/mL)			Concentration of K1-18 (µg/mL)		
Date of blood collection	800	400	0	800	400	0
2009.11.02^a^	<40	<40	40	<20	<20	20
2009.11.09	80	320	320	<40	<40	40
2009.11.17	640	640	1280	40	40	40
2009.11.23	320	640	2560	40	40	40
2009.12.07	320	640	2560	40	40	80
2009.12.14	320	320	2560	<40	40	80

a Serum before vaccination.

## Discussion

Historically, it has been believed that vaccines remain the most reliable method to control seasonal epidemics of influenza. However, the mode of response against influenza virus infection is very heterogeneous among the human population. Since we experience an outbreak of flu almost every year in Japan, most of the people may easily have opportunities to be infected by influenza viruses. Nevertheless, there are many people who appear to be resistant to the seasonal epidemics without vaccination. On the other hand, there are many people who are vaccinated almost every year but contract influenza quite often. In the present study we analyzed the repertoire of neutralizing Abs against H1N1 viruses in a flu-resistant person. We also tried to analyze the effects of vaccination with formalin-treated virus particles on the Ab repertoire under the conditions without effects of natural infection with live viruses. This experiment can be performed only at the outbreak of a pandemic. Since people are naive to the pandemic virus, we can perfectly exclude the possibility that the examinee has been naturally infected with the virus used as vaccine.

We obtained very simple results. Only two types of Abs were isolated with no exceptions. The first type is the products of B cells newly induced through vaccination and the second type is the products of long-lived memory B cells established before vaccination. While there should be more clones that we have overlooked in this screening, it is unlikely that we have specifically overlooked clones with characteristics very different from those listed in Figure 2. For example, Abs that bind to HA of seasonal H1N1 virus but not to HA of pandemic virus were not isolated in the screening 4 (before vaccination, with seasonal virus), and all of the 16 clones were the second type of Abs. Since the library used in this screening was constructed from B cells before vaccination, expansion of the first type of B cells through vaccination did not influence the characteristics of clones isolated from the screening 4. Thus, the number of B cells that produce Abs that bind to the globular head of HA of seasonal H1N1 virus should have been at a low level, even if they existed. This conclusion is further supported by the following observation. In the serum before vaccination, the virus-neutralizing activity against seasonal H1N1 virus examined by focus reduction assay was at a detectable level, but the HI activity was not detected (Table 1). Since the surrounding regions of the sialic acid-binding pocket are immunogenically very potent, B cells producing Abs that bind to the epitopes could be preferentially generated. Therefore, these results should have reflected the selection mechanism of long-lived memory cells.

Our results also indicate that majority of the memory cells present in the donor's body produce Abs with similar characteristics, that is, they utilize the *1-69* V_H_ gene, bind to the stem of HA, and neutralize both H1N1 and H5N1 viruses. They are major players in protection against H1N1 virus infection in the donor's body. We analyzed a person who had experienced influenza disease in their youth but never suffered from this disease afterwards. Since he has never been vaccinated against influenza, the Ab repertoire formed in his body was generated only by the effects of infection with live viruses. This observation is consistent with the following hypothesis. In our previous study [8], we analyzed the repertoire of neutralizing Abs against H3N2 viruses in a donor born in 1960. Many anti-HA Abs were isolated, and they were divided into three major groups with three distinct strain specificity: 1968–1973, 1977–1993 and 1997–2003. While five sites, such as A, B, C, D, and E, located on the globular head of HA have been identified as neutralizing epitopes [18], [19], most of the clones that neutralize the 1977–1993 strains bound to site C [20]. Between 1977 and 1993, many mutations were introduced into sites A and B, but no mutations were introduced into site C. In order to explain these observations, we proposed the following hypothesis [20]. After a set of B cells producing Abs that can neutralize the viruses are generated by infection and/or vaccination, they will take various courses under further stimulation with the Ags. Some B cells disappear but others remain as memory cells. Furthermore, there should be long-lived memory cells and short-lived memory cells. Humans who experience an outbreak of flu almost every year have opportunities to be infected by novel influenza viruses that have drifted away from previous viruses. Some memory cells produce Abs that are able to neutralize the novel viruses, but others produce Abs that cannot neutralize them. They would be selected through the presence or absence of stimulation with the Ags. According to this hypothesis, the stability of the epitope would greatly affect the fate of memory B cells. As already shown by other groups [15], [16], the epitope recognized by 1-69Abs is extremely stable among group 1 viruses. Thus, the cells producing 1-69Abs were selected as long-lived memory cells.

We analyzed only one person in this study. Therefore, the observations described in this paper should not be generalized at present. However, all humans should have potential for generating 1-69Abs that can broadly neutralize group 1 viruses, since only the *1-69* V_H_ gene is required for producing a broadly neutralizing Ab without participation of the V_L_ domain in forming the Ag-binding site [15], [16], and furthermore, the requirements of the CDR3 sequence in V_H_ for binding to the HA stem appeared to be limited [15], [16]. Thus, we propose that the resistance of people against influenza infections is explained by acquisition of the ability to produce Abs that bind to the stable epitope and that many people show the resistance against influenza since humans are able to easily generate 1-69Abs that neutralize all of group 1 viruses. The present study gave the example only for resistance against group 1 viruses. If this is the case, humans should also have abilities to generate Abs that can neutralize all H3 viruses. However, there have been few papers reporting isolation of broadly neutralizing Abs against H3 viruses from humans. CR8020 reported by Ekiert *et al.* [21] showed broad neutralizing activity against group 2 viruses including H3, and it binds to the HA stem distinct from the epitope recognized by 1-69Abs. F16 reported by Corti *et al.* [22] neutralized both group 1 and group 2 viruses, and it binds to a conserved epitope in the F subdomain of HA. In our previous studies [8], [14], [20], we analyzed B lymphocytes collected from three donors born in 1944, 1960, and 1974 and revealed the repertoire of neutralizing Abs against H3 viruses. As summarized above, the Abs produced in two donors born in 1960 and 1944 were divided into three major groups with three distinct strain specificities: 1968–1973, 1977–1993 and 1997–2003. On the other hand, the Abs in the donor born in 1974 were composed of only four different types. Two of them showed narrow strain specificity: most strongly bound to the 1973 strain, and bound to the 1997 to 2003 strains. The other two types showed extremely broad strain specificity: bound to all of H3 strains, and bound strongly to all of H3 strains, moreover, weakly to group 1 viruses. These observations could be interpreted as follows: since he obtained B cells producing Abs that neutralize all of H3 viruses, the B cells producing Abs that specifically neutralize the viruses present between 1980 and 1995 disappeared.

How to select the B cells that produce Abs binding to the stable epitope as long-lived memory B cells would be explained by the following observations. HI activity against H1N1 pandemic virus started to appear in the serum 2 weeks after the vaccination. On the other hand, in the case of virus-neutralizing activity measured by focus reduction assay, the activity against H1N1 pandemic virus already started to increase 1 week after vaccination and reached a plateau 2 weeks after vaccination. When viruses infect a person, memory B cells that produce Abs with the ability to neutralize the infected viruses are stimulated to grow earlier than the birth of B cells newly induced by infection with the viruses.

While further experiments are required to examine whether Abs produced in the type 2 clones are strong enough for preventing infection with future pandemics caused by highly pathogenic avian influenza (HPAI) H5N1 virus, we may expect that the presence of type 2 clones as memory cells could be helpful for preventing expansion of the pandemic viruses in their bodies. Thus, we propose that the strategy for protection against the H5N1 pandemic should be designed according to the immunological profile of each individual. Presence of anti-HA Abs utilizing the *1-69* V_H_ gene could be a useful indicator for presence of broadly neutralizing Abs and anti-idiotypic Abs against 1-69Abs could be the reagent.

## Supporting Information

Figure S1 **Amino Acid sequences of VH fragment of representative clone in each group.**
(TIF)

Figure S2 **Nucleotide sequences for CDR3 region of representative clone each group.**
(TIF)
