# Supplementary figures and images for: Two Types of Antibodies Are Induced by Vaccination with A/California/2009pdm Virus: Binding near the Sialic Acid-Binding Pocket and Neutralizing Both H1N1 and H5N1 Viruses
Source: PLoS One. 2014 Feb 5;9(2):e87305. doi: 10.1371/journal.pone.0087305 (PMC3914828; doi:10.1371/journal.pone.0087305)

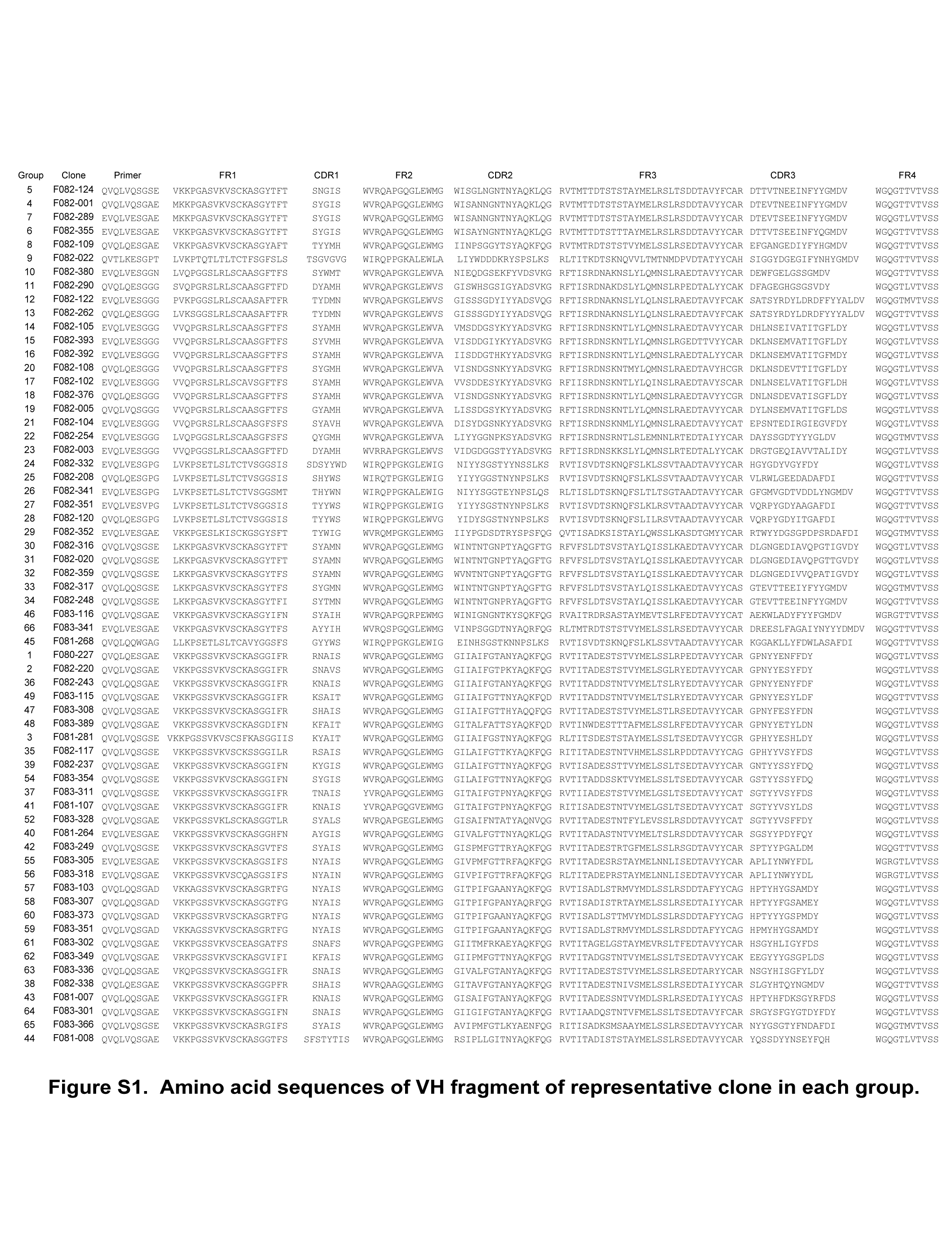

Supplement: Figure S1 — Amino Acid sequences of VH fragment of representative clone in each group. (TIF) [file pone.0087305.s001.tif]

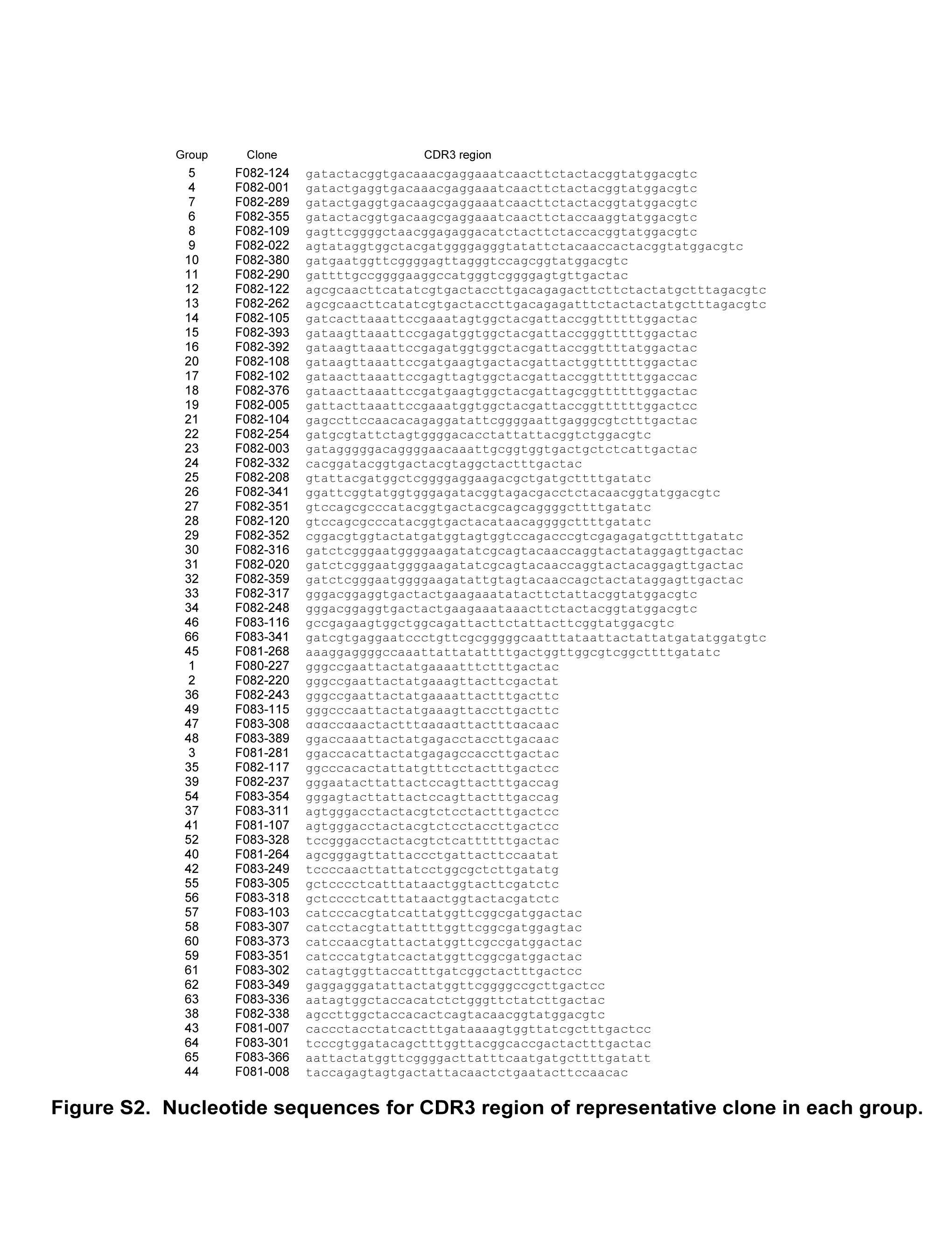

Supplement: Figure S2 — Nucleotide sequences for CDR3 region of representative clone each group. (TIF) [file pone.0087305.s002.tif]
